# Optimizing pharmacogenomic decision-making by data science

**DOI:** 10.1371/journal.pdig.0000451

**Published:** 2024-02-08

**Authors:** Amir M. Behdani, Jessica Lai, Christina Kim, Lama Basalelah, Trey Halsey, Krista L. Donohoe, Dayanjan Wijesinghe

**Affiliations:** Department of Pharmacotherapy and Outcomes Science, Virginia Commonwealth University School of Pharmacy, Richmond, Virginia, United States of America; Johns Hopkins Medicine, UNITED STATES

## Abstract

Healthcare systems have made rapid progress towards combining data science with precision medicine, particularly in pharmacogenomics. With the lack of predictability in medication effectiveness from patient to patient, acquiring the specifics of their genotype would be highly advantageous for patient treatment. Genotype-guided dosing adjustment improves clinical decision-making and helps optimize doses to deliver medications with greater efficacy and within safe margins. Current databases demand extensive effort to locate relevant genetic dosing information. To address this problem, Patient Optimization Pharmacogenomics (POPGx) was constructed. The objective of this paper is to describe the development of POPGx, a tool to simplify the approach for healthcare providers to determine pharmacogenomic dosing recommendations for patients taking multiple medications. Additionally, this tool educates patients on how their allele variations may impact gene function in case they need further healthcare consultations. POPGx was created on Konstanz Information Miner (KNIME). KNIME is a modular environment that allows users to conduct code-free data analysis. The POPGx workflow can access Clinical Pharmacogenomics Implementation Consortium (CPIC) guidelines and subsequently be able to present relevant dosing and counseling information. A KNIME representational state transfer (REST) application program interface (API) node was established to retrieve information from CPIC and drugs that are exclusively metabolized through CYP450, and these drugs were processed simultaneously to demonstrate competency of the workflow. The POPGx program provides a time-efficient method for users to retrieve relevant, patient-specific medication selection and dosing recommendations. Users input metabolizer gene, genetic allele data, and medication list to retrieve clear dosing information. The program is automated to display current guideline recommendations from CPIC. The integration of this program into healthcare systems has the potential to revolutionize patient care by giving healthcare practitioners an easy way to prescribe medications with greater efficacy and safety by utilizing the latest advancements in the field of pharmacogenomics.

## Author summary

Our team has developed a new program called Patient Optimization Pharmacogenomics (POPGx) to help healthcare providers efficiently determine the best pharmacogenomic medication recommendations for their patients. Pharmacogenomic medication selection and dosing recommendations improve patient safety and therapy efficacy by taking the patient’s genotype into account. This process is currently time-consuming and cumbersome for healthcare providers, which can result in human-error and decreases overall pharmacogenomic utilization. POPGx was built using the Konstanz Information Miner (KNIME) which is a modular, low-code environment that allows for rapid data analysis. The program utilizes Clinical Pharmacogenomics Implementation Consortium (CPIC) guidelines to provide relevant dosing and counseling information for each metabolizer type and allows healthcare providers to input a patient’s metabolizer gene, genetic allele data, and medication list. Our program is designed to streamline the pharmacogenomic therapy selection healthcare providers conduct for their patients. The goal of POPGx as demonstrated herein is to improve patient care by increasing medication safety and efficacy.

## Introduction

Pharmacogenomics refers to the application of patient genomic information to optimize medication selection and dosing and is a form of precision medicine that has grown exponentially in the past 50 years.[1] A relationship between genetic polymorphism and medication metabolism has been well established by multiple studies, such as in the case of the cytochrome P450 (CYP) enzymes family. DNA sequencing elucidates likely genotypes of many enzymes, including CYPs. Enzymes play a decisive role in drug metabolism, influencing both treatment efficacy and the potential for adverse drug reactions. Variations in alleles of enzyme genes can alter enzyme expression levels in vivo, ultimately affecting drug metabolism in the body. By utilizing pharmacogenetics, healthcare providers can predict and optimize treatment outcomes and patient safety [1].

Data science is a broad interdisciplinary field that involves the use of scientific methods, systems, and algorithms to guide the extraction of information and insights from data. It involves the use of various statistical and computational techniques to analyze and interpret data, and to predict and extrapolate decisions based on that data [2]. It also involves the creation of visualization and dashboards to communicate insights to stakeholders. Furthermore, the availability of health information has increased exponentially over the decades. Digital health programs can simplify access and understanding of the expansive amount of health information available in databases to bridge the understanding of complex topics between provider and patient [3,4]. The ultimate goal of data science in healthcare is to identify patterns and trends in medical data and turn them into actionable insights that can guide decision-making processes, ultimately leading to improved patient care. Patient care can be improved by earlier detection of diseases and optimal treatment with minimal delay to enhance life quality and expectancy. Additionally, data science can help make treatments more affordable by identifying more efficient and cost-effective methods [2].

Data science and pharmacogenomics are two fields that intersect in many ways. Genomic counseling is a process of communication between a healthcare provider and a patient that involves evaluating the risks, benefits, and limitations of genetic testing and other genomic information to help make informed decisions about health outcomes. The goal of this project is to utilize data science to facilitate personalized medicine by identifying the genetic factors that contribute to a patient’s specific condition. This information can be used to develop targeted treatments that are more likely to be effective for a particular patient, based on their genetic makeup. Using data science techniques, such as statistical analysis and machine learning, a data scientist can analyze patient data to identify genetic markers that can be used to predict a patient’s risk of developing certain side effects or drug interactions, as well as how a patient will respond to a particular medication. This information can then be used by healthcare providers to adjust the dosage of a medication to optimize its effectiveness and reduce the risk of side effects [5].

Pharmacogenetics can significantly affect an individual’s metabolic response to a medication which in turn will affect how the drug works in the body. An individual’s metabolic response is typically classified into four primary categories: ultrarapid metabolizers, extensive metabolizers, intermediate metabolizers, and poor metabolizers. These categories denote how efficiently a drug is metabolized by enzymes in the body. Ultrarapid metabolizers represent patients who metabolize drugs at a faster rate compared to the general population. For active drugs, this will lead to diminished therapeutic effects at standard dosages. For prodrugs, this will lead to increased drug levels in the bloodstream and a higher risk of adverse reactions. Extensive metabolizers form the reference group; people who fall into this category metabolize medications at a predictable rate and experience predictable therapeutic responses. Intermediate metabolizers metabolize drugs at a moderately reduced rate compared to extensive metabolizers. This means that for active drugs, this will lead to higher levels of the drug in the bloodstream with greater risk of side effects. For prodrugs, this will lead to suboptimal levels of the drug in the bloodstream. Lastly, poor metabolizers have significantly reduced or even absent enzyme activity and metabolize drugs much slower than the reference population. For active drugs, this can lead to very heightened drug levels in the bloodstream with a heightened risk of adverse reactions. For prodrugs, this can lead to little to no bioactivation, and thus very suboptimal levels of the drug in the bloodstream [6].

A significant challenge in incorporating pharmacogenetic data into standard clinical practice has been the absence of clear guidelines on adjusting medication use or dosage based on genetic test outcomes [7]. To address this, the Pharmacogenomics Knowledge Base (PharmGKB) collaborated with the National Institutes of Health (NIH) to establish the Clinical Pharmacogenetics Implementation Consortium (CPIC). Notably, CPIC’s guidelines emphasize the utilization of existing genetic test results rather than recommending which tests to order. This focus becomes more pertinent given the rising accessibility, decreasing costs of genetic tests, and the emergence of direct-to-consumer testing options.

The current approach to making pharmacogenetics-guided medication selection and dosing is through CPIC. Healthcare providers can use CPIC to optimize treatment selection and dosing. CPIC guidelines are standardized, peer-reviewed, evidence-based, and are freely available to the public [8]. PharmGKB is a comprehensive online database detailing gene-drug associations and genotype-phenotype relationships. In collaboration with PharmGKB, CPIC publishes guidelines based on these associations [9].

CPIC stands as an invaluable resource that aids in interpreting results and tailoring medication regimens. However, its user interface proves to be a challenge for many. Navigating the platform often directs users to dense scientific articles that require time-consuming analysis and interpretation. This not only is time-intensive but also increases the potential for error, as users may inadvertently misinterpret or overlook crucial information, especially when sifting through details for multiple drugs and gene abnormalities. Despite these challenges, many professionals still resort to manually searching CPIC, given the lack of a more efficient alternative. Our tool aims to simplify this process, extracting and presenting only the relevant information, thus streamlining the workflow and minimizing the risk of errors.

To address concerns with the current approach of using gene/drug guideline consortiums, Patient Optimization Pharmacogenomics (POPGx) was constructed. POPGx is an automated program that provides a straightforward user-interface for providers to input patient-specific genetic allele data, enzymes of interest, and medications to retrieve clear dosing recommendations. The objective of this paper is to describe the development of POPGx and its goal to create an easier to use approach for healthcare providers to determine pharmacogenomic dosing recommendations for patients taking multiple medications.

## Materials and Methods

POPGx, unlike the current gene/drug guideline consortium searching method, allows for multiple drugs in a patient’s regimen to be inputted into the system at one time. It retrieves and displays pertinent dosing recommendations in a centralized manner. The goal is to decrease the number of errors that can occur from the current tedious method of searching consortiums one medication at a time and having to navigate multiple guidelines. Healthcare providers can then easily interpret patients’ pharmacogenetic testing in relation to their patient’s medication to determine the correct dosing for efficacy and patient safety with POPGx.

POPGx was developed on the Konstanz Information Miner (KNIME) interface. The KNIME interface is an open-source modular environment which allows code-free data analysis to users by visual assembly and interactive execution of a data pipeline [10]. In this platform, individual tasks are represented by nodes that can be used to access, merge, analyze, and visualize data. Nodes can be used for integration of data from the outside sources if the data is publicly available. Since CPIC has its database and application program interface (API) available to the public, all the data can be accessed through a KNIME rest API node.

**Fig 1:**
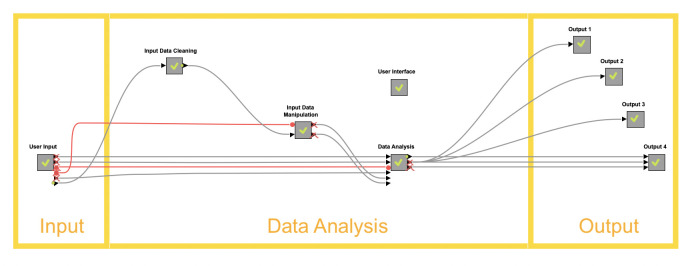
An illustration of the overall setup of CPIC in the KNIME analytics platform.

CPIC has a publicly available Representational State Transfer Application Programming Interface (REST API) [9]. This allows users to connect applications to the CPIC database and retrieve information. The GET Request node in KNIME analytical platforms acts as a bridge connecting CPIC and the KNIME user interface. After establishing a connection between KNIME and CPIC, comprehensive data cleaning and aggregation took place to meet the application’s goals. Subsequently, a user-friendly application user interface was developed to meet the needs of providers and patients with minimal training. The workflow was later deployed on KNIME Server to allow users to use this application remotely on any device with internet access. See Fig 1.

## Results

POPGx features a straightforward user interface that adapts to the needs and medical expertise of its users. The program has an “Instructions” option to allow first-time users to quickly learn how to use POPGx. There are two main services POPGx provides: 1. Gene-Allele Consultation, and 2. Pharmacogenomics Dosing Recommendations.

### Gene-Allele Consultation

**Fig 2:**
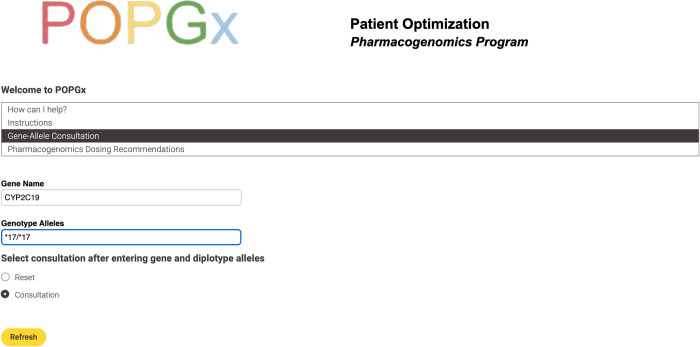
Gene-allele consultation inputs.

The “Gene-Allele Consultation” service is mainly tailored for patient use. This service calls for the user to input the gene name and the patient’s phenotype alleles to view the gene result and its corresponding consultation See Fig 2.

**Fig 3:**
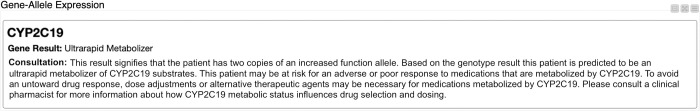
Gene-allele expression consultation based on inputs.

The gene result reports the patient’s likely metabolizer type based on their phenotype alleles. Gene results for metabolizer expression include: ultrarapid, extensive, intermediate, or poor. The corresponding consultation for that patient’s likely metabolizer gene result defines their metabolizer’s function status, associated risks and outcomes, and points the patient to consult a healthcare provider if needed. This consultation retrieval service increases patient accessibility to understanding the link between metabolizer type and medication therapy outcomes. See Fig 3.

**Fig 4:**
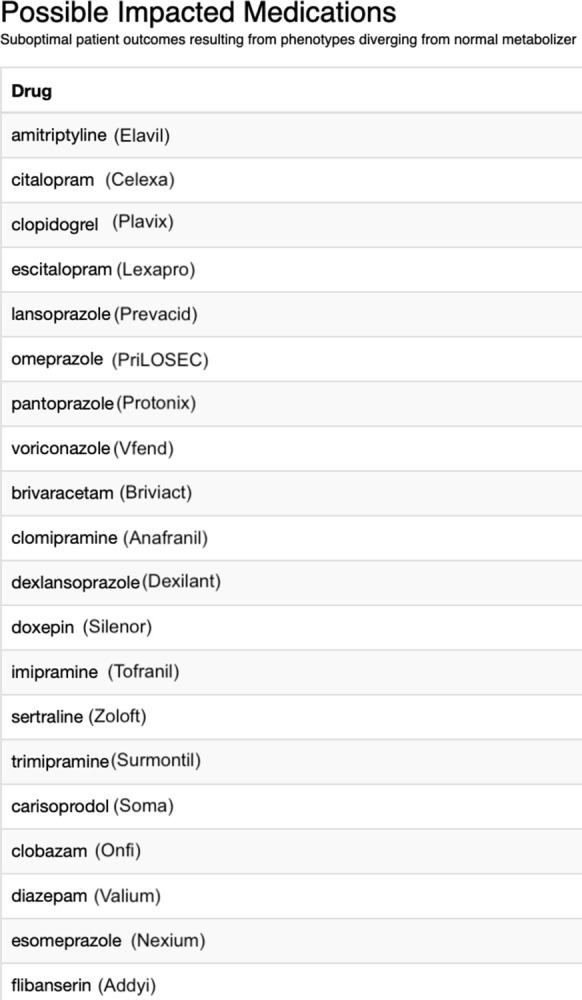
Possible impacted medications list.

Additionally, the “Gene-Allele Consultation” provides the user with a list of possible impacted medications related to the gene name and phenotype allele inputs. See Fig 4. This list of medications is related to suboptimal patient outcomes resulting from phenotypes diverging from the normal metabolizer. This curated list allows patients to quickly skim through and recognize any medication they may be taking. This at-a-glance list provides another layer for patients and providers to detect suboptimal therapy and enhance patient safety accordingly.

### Pharmacogenomics Dosing Recommendations

**Fig 5:**
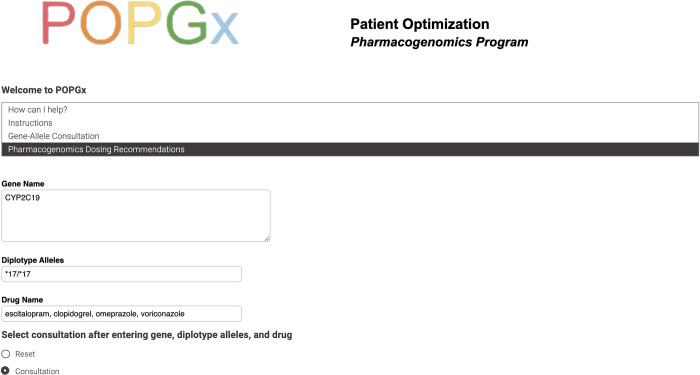
Pharmacogenomics dosing recommendations inputs.

The “Pharmacogenomics Dosing Recommendation” service is customized for healthcare provider use. This service calls for users to input gene name, diplotype alleles, and drug name to retrieve corresponding dosing recommendations. See Fig 5.

**Fig 6:**
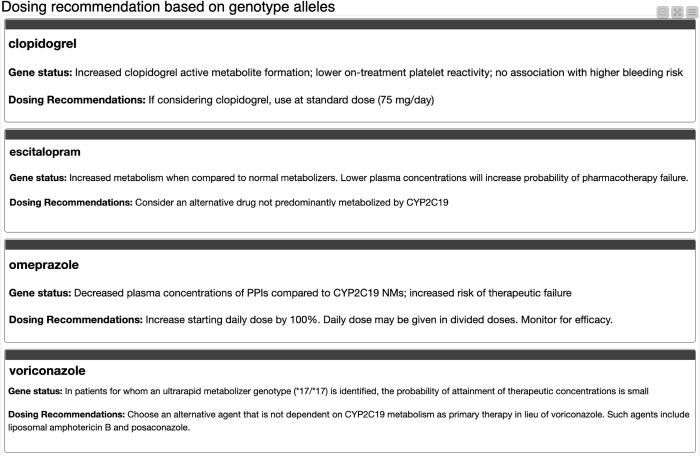
Dosing recommendations based on inputs.

The “Pharmacogenomics Dosing Recommendation” service defines the patient’s gene status based on the above inputs. See Fig 6. The gene status describes the clinical outcome if the patient was continued on normal dosing without regard to mutant metabolizer expression. Subsequently, it provides a clear dosing recommendation with regards to the patient’s metabolizer type and corresponding medication. The service also allows a provider to input multiple medications into a patient’s therapy regimen at one time. This unique option–inputting multiple medications instead of looking up one medication at a time–streamlines the process providers must go through to retrieve dosing recommendations, which subsequently may lead to less medication errors and boost patient safety.

The number of genetic data points required to be entered will vary among individuals, as it greatly depends on the specific medication list of each patient. Not all medications have pharmacogenomic implications, meaning that not every drug would necessitate an associated genetic data point. Furthermore, the concept of multiplicity plays a role in this process. If a patient is prescribed several medications that interact with the same implicated gene, it could potentially reduce the number of data points needed and the time spent inputting this information.

## Discussion

**Fig 7:**
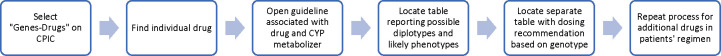
Gene/Drug guideline consortium process.

The current approach for making pharmacogenomic-based therapy selection and dosing modification is tedious. Online gene/drug guideline consortiums, such as CPIC, require healthcare providers to search for a patient’s drug individually to retrieve the guideline associated with the drug’s metabolizer of concern. From there, a provider would have to match the patient’s diplotype to the likely phenotype. Then, the phenotype would be matched to a dose recommendation in a separate table further down the guideline. This entire process only accounts for one drug in a patient’s medication regimen, and the provider would have to repeat this process for each drug in a patient’s medication regimen to optimize medication efficacy and patient safety (see Fig 7).

If a patient is on multiple medications or has multiple genes that are not heterogenous metabolizers, the provider has to reiterate the process mentioned in Fig 7 multiple times. The process is time-consuming and will potentially increase the risk of medical errors. However, the Patient Optimization Pharmacogenomics (POPGx) program does this process fully automated using the most updated clinical data from CPIC. The only requirement would be entering the patient-related genetic data by the user, and the program will provide results in seconds.

Currently, it is time intensive to search guidelines to determine a patient’s correct pharmacogenetic dosing if they have a variation in their alleles. The POPGx workflow was created to improve this process. POPGx provides comprehensive dosing recommendations in a time-efficient manner, streamlining the prescribing process for healthcare providers while also allowing patients to view their pharmacogenomic data in regard to their medications.

POPGx has been mainly experimented with and employed by pharmacists and pharmacy students, with its primary application focused on educational aspects. This tool is useful for simulating patient scenarios for pharmacy or medical students. It helps pharmacy students become more comfortable and familiar with the concept of pharmacogenomics and how it impacts medication prescribing and dosage. Feedback from pharmacists and students indicates that the tool has been invaluable in substantially cutting down the time spent on tasks and mitigating potential errors. Although the tool’s application with patients and non-pharmacist providers remains unexplored, early indications from its current users highlight its potential for streamlining operations whilst also ensuring accuracy.

The design and interface of the tool are evidently user-friendly and efficient. For a typical patient, the process is straightforward, demanding only necessary information to present desired results. The streamlined and efficient data input process enhances user engagement for both patients and for health care professionals.

The advantage of using KNIME is due to modularity and the ease of use for those that do not have any data science background. Since the majority of healthcare providers do not receive comprehensive training in data science, using a platform like KNIME bridges the gaps in healthcare with the use of code-free data science. Subsequently, it allows users to do rapid prototyping with minimal data science background. For instance, this program was developed from conception to utilization in less than 15 hours for a healthcare hackathon. Moreover, different workflows built through KNIME can either be used independently or they could get connected to one another to offer a wider range of capabilities. For instance, an area of improvement could be connecting this workflow to dosing calculator programs that were previously built through KNIME to execute multiple functions simultaneously. KNIME also offers KNIME Server, an enterprise-grade solution for Advanced Analytics workloads such as sharing workflows and executing workflows [11]. This allows KNIME workflows to be used by any user without even using the KNIME software.

A current alternative for optimizing pharmacogenomic patient health outcomes is DNA tests with pharmacogenomic reports, such as GeneSight, that patients can utilize through a provider to determine how their body metabolizes their medications [12]. While services like GeneSight are individualized per patient, they are still very limited in use, difficult to order, and are inefficient in returning results; furthermore, analyses are confined to mental health disease states and often only include a patient’s pharmacokinetic interactions with the drugs in question, and do not include any dosing recommendations [12]. Application of these products is also not generalizable to many patients and is expensive [12]. POPGx works around these limitations by returning information quickly without the need for a proxy, and by providing all necessary details to the provider and the patient, including a regimen and pharmacokinetics. POPGx is also applicable to hundreds of drugs and disease states, not solely restricted to psychotropics.

While POPGx can retrieve valuable pharmacogenomic information to healthcare providers and patients alike, it has some limitations. Currently, it is completely reliant on the CPIC database, which makes it vulnerable to CPIC’s system interruptions or delays in upgrades. One possible solution to this would be to incorporate other pharmacogenomic databases to allow users to verify information from multiple sources. However, it is noteworthy to mention that this tool aims to refine the prevailing method of pharmacogenomic data retrieval. Currently, providers often resort to labor-intensive procedures, such as manually sifting through guideline articles to obtain pertinent data. Even renowned platforms like CPIC merely guide users to specific publications, which they then have to study to extract the necessary information. This not only consumes an exorbitant amount of time but also heightens the risk of errors. In this context, our tool presents a promising avenue to revolutionize the existing system, making it more efficient and reliable.

Additionally, POPGx does not facilitate the direct downloading of genetic and medication data. This limitation stems from the fact that the tool is not yet integrated with any Electronic Health Record (EHR) systems. However, plans for the tool’s enhancement envision its linkage to EHR systems, allowing for automatic data population using patient information that will allow healthcare providers to easily access pharmacogenomic recommendations based on patient profiles. Furthermore, POPGx is currently only available on computers that meet all the application requirements, but it could become more accessible in future updates via web or mobile access.

To integrate the tool within the EHR, the plan encompasses the following steps: firstly, the specific integration needs of the tool with respect to the EHR’s existing functionalities need to be identified. Prioritizing data security and patient privacy, it is essential to ensure full compliance with relevant regulatory standards, such as HIPAA. Subsequently, the most suitable data integration methodology for this purpose, considering standards like HL7 or FHIR, needs to be chosen. Given the intricacies of data flow, it’s essential to establish a robust error-handling mechanism to preemptively address any discrepancies or issues. Before full-scale deployment, rigorous testing of the integrated system should be undertaken to ascertain its reliability and efficiency. Lastly, comprehensive training for end-users needs to be provided to ensure they can effectively leverage the integrated tool within the EHR environment.

Currently, POGPGx has been primarily tested and utilized by pharmacists and pharmacy students for educational purposes. Its main objective during this phase has been to enhance the efficiency and effectiveness of pharmacogenomics counseling. While there is no data from a commercial website to cite, the feedback and results from these educational applications have been promising. It is important to note that direct application in real-world clinical settings, with patients directly benefiting from the tool or healthcare providers assisting patients with it, remains a future direction. We are optimistic about its potential and look forward to extending its reach to provide tangible benefits in real-world practice. Additionally, by publishing our findings and the details of our tool, we hope to inspire other innovators in the field to develop similar programs. The goal is not only to further enhance the current landscape of tools available but also to address the time-consuming processes and minimize the potential for user error inherent in existing tools.
